# Complete mitochondrial genome sequence of *Urechis caupo*, a representative of the phylum Echiura

**DOI:** 10.1186/1471-2164-5-67

**Published:** 2004-09-15

**Authors:** Jeffrey L Boore

**Affiliations:** 1 Evolutionary Genomics Department, DOE Joint Genome Institute and Lawrence Berkeley National Laboratory, 2800 Mitchell Drive, Walnut Creek, CA, USA; 2 Department of Integrative Biology, University of California, Berkeley, CA, USA

**Keywords:** mtDNA, evolution, gene rearrangement, annelid, strand skew

## Abstract

**Background:**

Mitochondria contain small genomes that are physically separate from those of nuclei. Their comparison serves as a model system for understanding the processes of genome evolution. Although hundreds of these genome sequences have been reported, the taxonomic sampling is highly biased toward vertebrates and arthropods, with many whole phyla remaining unstudied. This is the first description of a complete mitochondrial genome sequence of a representative of the phylum Echiura, that of the fat innkeeper worm, *Urechis caupo*.

**Results:**

This mtDNA is 15,113 nts in length and 62% A+T. It contains the 37 genes that are typical for animal mtDNAs in an arrangement somewhat similar to that of annelid worms. All genes are encoded by the same DNA strand which is rich in A and C relative to the opposite strand. Codons ending with the dinucleotide GG are more frequent than would be expected from apparent mutational biases. The largest non-coding region is only 282 nts long, is 71% A+T, and has potential for secondary structures.

**Conclusions:**

*Urechis caupo *mtDNA shares many features with those of the few studied annelids, including the common usage of ATG start codons, unusual among animal mtDNAs, as well as gene arrangements, tRNA structures, and codon usage biases.

## Background

Mitochondrial genomes are physically separate from the nuclear genome. For animals, they are typically circular with a compact arrangement of an identical set of 37 genes [1]. For some animals, all genes are on the same strand, whereas for others they are divided between the two. The arrangement of these genes can remain stable for long periods of time; for example, human [2] and shark [3] mtDNAs have the same gene arrangement, and do those of fruit fly [4] and shrimp [5]. However, in other lineages, rearrangements have occurred much more rapidly. Many of the same processes that occur in large and complex nuclear genomes also take place in these diminutive genomes, so comparisons among mtDNAs can address general questions of genome evolution, but in a model system that is much more tractable for a large number of taxa.

Toward this end, this article describes the complete mtDNA sequence of the fat innkeeper worm, *Urechis caupo*, the first example from the phylum Echiura. Echiurans comprise about 150 species and are commonly called spoon worms because of the shape of their extensible proboscis. Unlike annelids, they have no overt segmentation, but they develop via trochophore larvae, very similar to those of annelids. *U. caupo *is a pink, sausage shaped worm that lives in U-shaped burrows in the mud or sand of the intertidal or subtidal zones. Unlike other echiurans, it feeds on plankton by filtering using an elaborate mucus net.

## Results and discussion

### Gene content and organization

**Figure 1:**
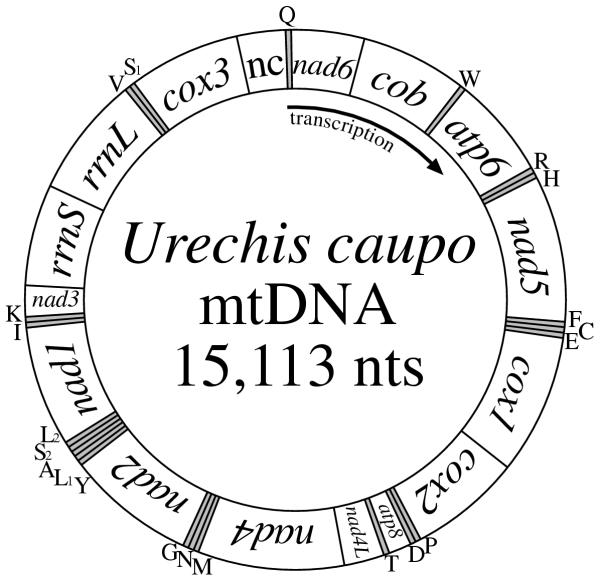
**Mitochondrial gene map of the echiuran *Urechis caupo. ***All genes are transcribed from the same DNA strand. Scaling is only approximate. Genes are designated by standard nomenclature except for tRNAs, which are identified only by the one-letter code for the corresponding amino acid, with the two serine and two leucine tRNAs differentiated by numeral as identified in Fig. 3. "nc" indicates the largest non-coding regions; it may be that transcription initiates here, but this is not known.

The mtDNA of *Urechis caupo *is 15,113 nts in length (GenBank accession number **AY619711**) and contains the same 37 genes found for nearly all animal mtDNAs [see ref. [1]]. All genes are transcribed from the same strand (Fig. 1), as is the case for the two studied annelid mtDNAs, the polychaete *Platynereis dumerilii *[6] and the oligochaete *Lumbricus terrestris *[7] and for several other animal mtDNAs. The arrangement of the genes is substantially similar to those of the two annelids, and shares short regions of similarity with several other mtDNAs, as can be seen in Table 1.

**Table 1:** Mitochondrial gene arrangement identities found in pairwise comparisons between *Urechis caupo *and various animals. Full taxon names are given here for the annelids *Lumbricus terrestris *and *Platynereis dumerilii*, the mollusks *Katharina tunicata*, *Loligo bleekeri*, *Cepaea nemoralis*, and *Mytilus edulis*, the brachiopods *Terebratulina retusa *and *Terebratalia transversa*, the platyhelminths *Fasciola hepatica*, *Taenia crassiceps*, *Echinococcus multilocularis*, and *Hymenolepis diminuta*, the arthropods *Drosophila yakuba*, *Anopheles gambiae*, *Artemia franciscana*, *Daphnia pulex*, *Apis mellifera*, *Locusta migratoria*, *Ixodes hexagonus*, *Rhiphicephalus sanguineus*, *Limulus polyphemus *and *Lithobius forficatus*, the nematodes *Trichinella spirallis*, *Onchocerca volvulus*, *Meloidogyne javanica*, *Ascaris suum*, and *Caenorhabditis elegans*, the echinoderms *Arabacia lixula*, *Asterina pectinifera*, *Paracentrotus lividus*, *Strongylocentrotus purpuratus*, and *Florometra serratissima*, the hemichordate *Balanoglossus carnosus*, and the chordate *Branchiostoma floridae *along with the gene order most typical for vertebrates. Complete citations can be found in Boore (1999) or updated by following the "Evolutionary Genomics" link at . Contiguous gene arrangements are separated by a comma; a slash indicates a gap containing one or more unrelated genes.

*L. terrestris *and *P. dumerilii*	*cox3*, *trnQ*, *nad6*, *cob*, *trnW*, *atp6*, *trnR*, *trnH*, *nad5*, *trnF*/*trnL2*, *nad1*, *trnI*, *trnK*, *nad3*/*trnT*, *nad4L*, *nad4*
*L. terrestris *but not *P. dumerilii*	*trnL1*, *trnA*, *trnS2*, *trnL2*/*trnD*, *atp8*
*K. tunicata*	*trnL2*, *nad1*/*nad4L*, *nad4*/*trnH*, *nad5*, *trnF*
*L. bleekeri*	*nad6*, *cob*/*nad4L*, *nad4*/*nad5*, *trnF*/*trnD*, *atp8*
*C. nemoralis*	*trnL1*, *trnA*
*M. edulis*	*trnL2*, *nad1*/*trnT*, *nad4L*
*T. retusa*	*trnL2*, *nad1*/*nad4L*, *nad4*/*trnH*, *nad5*, *trnF*/*trnL1*, *trnA*/*trnD*, *atp8*/*cox1*, *cox2*
*T. transversa*	*trnP*, *trnD*
*F. hepatica*, *T. crassiceps*, *E. multilocularis*	*trnI*, *trnK*, *nad3*/*nad4L*, *nad4*/*trnY*, *trnL1*/*trnS2*, *trnL2*
*H. diminuta*	*trnI*, *trnK*, *nad3*/*nad4L*, *nad4*/*trnY*, *trnL1*
*D. yakuba*, *A. gambiae*, *A. franciscana*, *D. pulex*	*nad6*, *cob*/*nad4L*, *nad4*/*trnH*, *nad5*, *trnF*/*trnD*, *atp8*
*A. mellifera*, *L. migratoria*	*nad6*, *cob*/*nad4L*, *nad4*/*trnH*, *nad5*, *trnF*
*I. hexagonus*, *R. sanguineus*, *L. polyphemus*, *L. forficatus*	*nad6*, *cob*/*trnL2*, *nad1*/*nad4L*, *nad4*/*trnH*, *nad5*, *trnF*/*trnD*, *atp8*/*cox1*, *cox2*
*T. spirallis*	*nad6*, *cob*/*nad4L*, *nad4*/*trnH*, *nad5*, *trnF*/*trnR*, *trnH*/*trnL1*, *trnA*/*trnD*, *atp8*/*cox1*, *cox2*
*O. volvulus*	*trnP*, *trnD*
*M. javanica*	*trnN*, *trnG*
*A. suum*	NONE
*C. elegans*	NONE
*A. lixula*, *A. pectinifera*, *P. lividus *and *S. purpuratus*	*trnL2*, *nad1*, *trnI*
*F. serratissima*	*nad1*, *trnI*/*nad2*, *trnY*
*B. carnosus*	*trnL2*, *nad1*/*nad4L*, *nad4*
*B. floridae *and the typical vertebrate arrangement	*trnL2*, *nad1*, *trnI*/*nad4L*, *nad4*

### Base composition and codon usage

The *U. caupo *mtDNA is 62% A+T, about the same as for annelid mtDNAs (64% and 62% for *P. dumerilii *and *L. terrestris*, respectively). As is typical, all homodinucleotides and homotrinucleotides are greatly over represented relative to a random distribution and CG is the least frequent dinucleotide, both in absolute number and in the ratio of observed to expected. The gene-coding strand has a strong skew against G vs. C but about equal amounts of A vs. T; G-skew ([G-C]/[G+C]) is – 0.24 and T-skew ([T-A]/[T+A]) is – 0.016 [8]. These values show no striking variation across the length of the mtDNA. Codon usage (Table 2) reflects these values, with those ending in A or T being most frequent. In all cases except for two, where they are synonymous, NNC codons are in greater abundance than NNG codons, as is consistent with the coding strand being rich in C relative to G. The two exceptions are CGG and GGG codons, which are each in greater abundance than their respective synonyms, CGC and GGC. This invites the speculation that there is something favored about the GG dinucleotide created when G appears in the second codon position. However, this is not consistently seen, since in the remaining case, AGC codons outnumber AGG codons two-to-one. This effect has been shown to be very strong for codon usage pattern of the mtDNA of the brachiopod *Terebratalia transversa *[9].

**Table 2:** Codon usage in the 13 protein-encoding genes of the *Urechis caupo *mitochondrial genome. The total number of codons is 3722. The anticodon of the corresponding tRNA gene is shown in parentheses below each amino acid designation. Stop codons are not included in this analysis.

Amino acid	Codon	N	%	Amino acid	Codon	N	%
Phe (F)	TTT	161	4.3%	Ser (S2)	TCT	108	2.9%
(GAA)	TTC	115	3.1%	(TGA)	TCC	65	1.7%
Leu (L2)	TTA	146	3.9%		TCA	74	2.0%
(TAA)	TTG	10	0.3%		TCG	3	0.1%
Tyr (Y)	TAT	42	1.1%	Cys (C)	TGT	18	0.5%
(GTA)	TAC	65	1.7%	(GCA)	TGC	11	0.3%
TER	TAA	---	---	Trp (W)	TGA	77	2.1%
	TAG	---	---	(TCA)	TGG	21	0.6%
Leu (L1)	CTT	105	2.8%	Pro (P)	CCT	72	1.9%
(TAG)	CTC	62	1.7%	(TGG)	CCC	44	1.2%
	CTA	224	6.0%		CCA	80	2.1%
	CTG	38	1.0%		CCG	6	0.2%
His (H)	CAT	33	0.9%	Arg (R)	CGT	6	0.2%
(GTG)	CAC	55	1.5%	(TCG)	CGC	5	0.1%
Gln (Q)	CAA	84	2.3%		CGA	46	1.2%
(TTG)	CAG	12	0.3%		CGG	9	0.2%
Ile (I)	ATT	200	5.4%	Thr (T)	ACT	76	2.0%
(GAT)	ATC	100	2.7%	(TGT)	ACC	91	2.4%
Met (M)	ATA	171	4.6%		ACA	93	2.5%
(CAT)	ATG	52	1.4%		ACG	5	0.1%
Asn (N)	AAT	61	1.6%	Ser (S1)	AGT	7	0.2%
(GTT)	AAC	65	1.7%	(TCT)	AGC	16	0.4%
Lys (K)	AAA	78	2.1%		AGA	62	1.7%
(TTT)	AAG	14	0.4%		AGG	8	0.2%
Val (V)	GTT	49	1.3%	Ala (A)	GCT	75	2.0%
(TAC)	GTC	28	0.8%	(TGC)	GCC	75	2.0%
	GTA	99	2.7%		GCA	122	3.3%
	GTG	18	0.5%		GCG	14	0.4%
Asp (D)	GAT	23	0.6%	Gly (G)	GGT	14	0.4%
(GTC)	GAC	37	1.0%	(TCC)	GGC	27	0.7%
Glu (E)	GAA	73	2.0%		GGA	127	3.4%
(TTC)	GAG	10	0.3%		GGG	36	1.0%

### Gene initiation and termination

Mitochondrial genes commonly use several alternatives to ATG as start codons. However, 11 of the 13 proteins coding genes of *U. caupo *mtDNA use ATG. The only exceptions are *cox1*, which uses GTG and *nad3 *which uses ATC. In the case of *cox1*, there is an in frame stop only three codons upstream and neither of the intervening codons is ATG. Also, this inference of starting on GTG specifies a set of amino acids well matched to those at the beginning of other Cox1 proteins. The situation for *nad3 *is nearly identical, with an in frame stop only four codons upstream and no intervening ATG codons. However, downstream of the inferred start are several ATA codons that can not be ruled out as alternatives. The commonality of using ATG as a start codon has also been noted for mitochondrial genes of four annelids, *Platynereis dumerilii *[6], *Lumbricus terrestris *[7], *Helobdella robusta *and *Galathealinum brachiosum *(previously considered to be of the phylum Pogonophora) [10] and a sipunculid, *Phascolopsis gouldii *[11].

**Figure 2:**
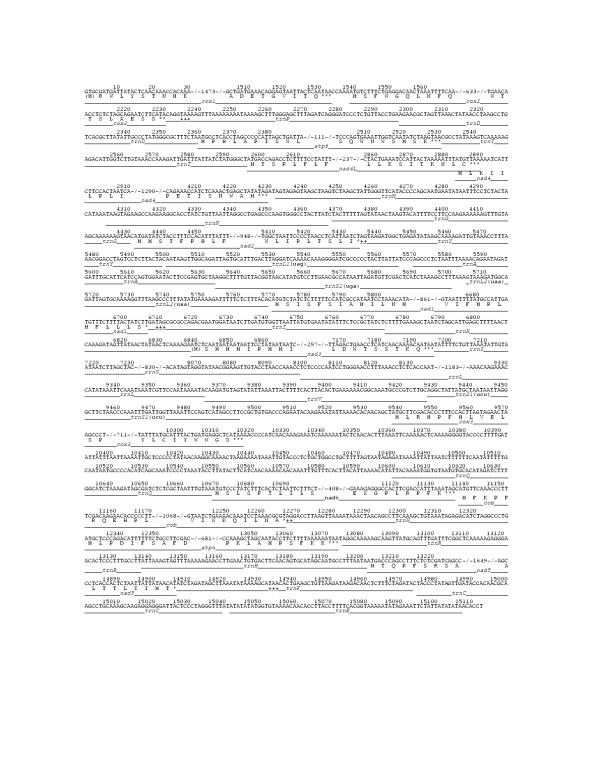
**A greatly abbreviated schematic of the sequence of *Urechis caupo *mtDNA. **In the interest of brevity, the middle portion of each large gene is omitted and replaced by a numeral indicating the number of nucleotides removed. Since all mitochondrial proteins are thought to initiate with formyl-methionine, an M is placed in parentheses at the first codon position of *cox1 *(GTG) and *nad3 *(ATC) to indicate nonconformity to the genetic code. Asterisks indicate inferred stop codons whether complete or abbreviated and plus symbols mark nucleotides that would form the first in frame, complete stop codon if genes instead overlap.

A complete stop codon without overlap of the downstream gene is found for all except *cox2*, *nad1*, *nad2*, *cob*, and *nad5 *(Fig. 2). In each of these cases, it appears that an abbreviated stop codon is generated by cleavage of a downstream tRNA from the polycistronic transcript, which is then completed to a TAA stop codon by polyadenylation. However, in two of these cases (*nad2 *and *cob*), a complete stop codon could be formed by including only the next two nucleotides, and two other cases (*nad1 *and *cox2*), there is an in frame stop codon just one or two codons downstream, respectively. It is not clear how gene overlaps could be resolved from a polycistronic transcript (assuming that the genes of this mtDNA are expressed in this way), but the presence of these stop codons seems beyond coincidence. It could be that they serve as a "back up" in case translation should begin in the absence of transcript cleavage.

### Transfer RNAs

**Figure 3:**
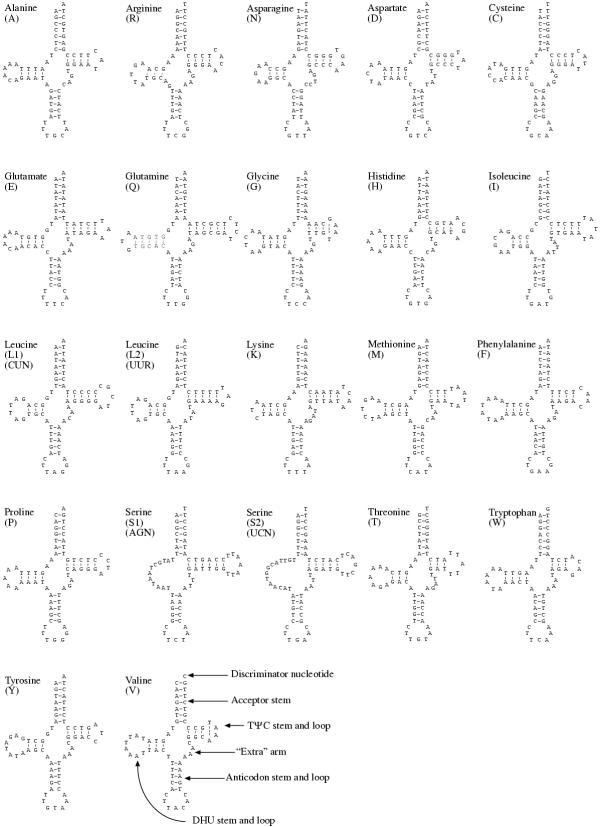
**The 22 inferred tRNA genes folded into the typical cloverleafstructures. **Nomenclature for tRNA substructures is indicated on tRNA(V).

Twenty-two regions can be folded into the typical cloverleaf structures of the expected set of tRNAs (Fig. 3). There are several mismatched nucleotide pairs within stems; nearly all of these are flanked by multiple G-C pairs, suggesting that they may provide compensatory stability for these arms. T precedes the anticodon and a purine follows it for all tRNAs. The two serine tRNAs lack potential for folding a DHU arm, as has been found for a number of other animal mtDNAs. There is an alternative folding possible for tRNA(S2) with a six-member anticodon stem and only one nucleotide separating the acceptor and DHU stems; this unusual folding has been found for the homologous genes of some mammals. tRNA(R) also does not have potential for a normally paired DHU arm, although there are three potential nucleotide pairs if two (rather than one) nucleotides were between the DHU and anticodon stems. However, this potential pairing could, alternatively, be a coincidence, with the DHU arm having no paired stem for this tRNA. Those with paired DHU arms have stems of three to five nucleotide pairs and loops of three to eight nts. All tRNAs have potential for stems of three to six nucleotide pairs for their TΨC arms with loops of three to seven nts. One of the tRNAs for serine has the anticodon TCT; although this is often found, the alternative of GCT is otherwise common.

### Ribosomal RNAs

As has been the case for all studied animal mtDNAs to date, two rRNA genes are identified, one for each of the small and large mitochondrial ribosomal subunits. Determining the precise ends of the rRNA transcript requires experimentation, but if it's assumed that they extend to the boundaries of the adjacent genes, then *rrnS *is 903 nucleotides and *rrnL *is 1266 nucleotides in length. These genes are arranged sequentially, but without an intervening tRNA gene as is otherwise commonly found.

### Non-coding regions

The largest non-coding region is only 282 nts long. The region is 71% A+T and contains one palindrome of an 11 nt sequence (TCAAAAGGGGT/ACCCCTTTTGA, with a slash indicating the center), but otherwise no large repeat elements. Obviously, this has potential for forming a stem-loop structure, and it may be significant that a short sequence a few nucleotides upstream, TCAAAA, has the potential for competing with this to form a short hairpin with the TTTTGA at the end of the palindrome. There has been previous speculation that regions with potential for competing, mutually exclusive hairpins may play a role in regulating transcription and/or replication [e.g. ref. [7]]. There are four other potential hairpins in this region with stems of 5–6 bp and loops of 5–17 nt. All four nucleotides occur in homopolymers with much greater frequency than expected by chance, often in runs of four or five. The second largest non-coding region is 43 nt between *trnS1 *and *cox3*. This has no repeat elements and the base composition is unremarkable. What role, if any, these sequences have in the regulation of transcription and/or replication awaits further study.

Aside from these 282 and 43 nt regions, there are only 36 total intergenic nucleotides scattered among 14 regions. In seven cases these are 2–6 nts long (CCAAA, AT, TCCC, TAAA, CATAAA, AT, and ACACCT). For the other seven cases, genes are separated by a single nucleotide, and in six of these, that nucleotide is a C. (The remaining case is a T.) The prevalence of C is consistent with the measured G-skew between the strands, although it is possible that this otherwise indicates some function of these nucleotides.

## Conclusions

This is the first description of a complete mitochondrial genome sequence of a representative of the phylum Echiura. The genome contains the same 37 genes most commonly found in animal mtDNAs. Many features are most similar to those found for annelid mtDNAs, including A+T content, use of protein initiation codons, size and potential secondary structures of the largest non-coding region, and the relative arrangement of many genes. As in annelids examined to date, all genes are found on the same DNA strand. As noted for brachiopod mtDNA, there is a preference for G nucleotides to appear in tandem, without obvious explanation. Further description and comparison of complete mtDNA sequences will continue to produce a picture of genome evolution, particularly once sampling includes representatives of each animal phylum.

## Methods

### Molecular techniques

A preparation of *Urechis caupo *total DNA was the kind gift of Eric Rosenthal. The entire mtDNA sequence was obtained using techniques detailed in [9]. Briefly, small fragments (450–710 nt) were amplified from *cox1*, *cob*, and *rrnS *using primer pairs HCO 2198/LCO 1490 [12], CytbF/CytbR [10], and 16SARL/16SBRH [13], respectively. The sequences of these fragments were determined using dye-terminator chemistry (PE Biosystems) on an ABI 377 automated DNA sequencer. Primers were then designed facing "out" from these fragments to amplify the intervening regions (~2.9 to ~8 Kb) using long-PCR protocols with rTth-XL polymerase (PE Biosystems) as in [9]. Sequences were determined from the ends of these long-PCR fragments, then internally by "primer walking". To ensure quality, all sequences were determined on both strands and base calls for all chromatograms were verified by eye.

### Gene annotation

Genes encoding rRNAs and proteins were identified by matching nucleotide or inferred amino acid sequences to those of *Lumbricus terrestris *mtDNA [7]. Since it is not possible to precisely determine the ends of rRNA genes by sequence data alone, they were assumed to extend to the boundaries of flanking genes. Each protein gene start was inferred as the eligible initiation codon nearest to the beginning of its alignment with homologous genes that does not cause overlap with the preceding gene. In five cases, an abbreviated stop codon was inferred where cleavage of a downstream tRNA from the transcript would leave a partial codon of T or TA, such that subsequent mRNA polyadenylation could generate a TAA stop codon. In each case an extension of this gene to the first in frame stop codon would cause overlap with the downstream tRNA. Genes for tRNAs were identified generically by their ability to fold into a cloverleaf structure and specifically by anticodon sequence.

## Abbreviations

*cox1*, *cox2*, *cox3*, cytochrome oxidase subunit I, II, and III protein genes; *cob*, cytochrome b gene; *atp6*, *atp8*, ATP synthase subunit 6 and 8 genes; *nad1*, *nad2*, *nad3*, *nad4*, *nad4L*, *nad5*, *nad6*, NADH dehydrogenase subunit 1–6, 4L genes; *trnA*, *trnC*, *trnD*, *trnE*, *trnF*, *trnG*, *trnH*, *trnI*, *trnK*, *trnL1*, *trnL2*, *trnM*, *trnN*, *trnP*, *trnQ*, *trnR*, *trnS1*, *trnS2*, *trnT*, *trnV*, *trnW*, *trnY*, transfer RNA genes designated by the one-letter code for the specified amino acid, with numerals differentiating cases where there are two tRNAs for the same amino acid.
